# Coated Blade Spray-Mass Spectrometry as a New Approach for the Rapid Characterization of Brain Tumors

**DOI:** 10.3390/molecules27072251

**Published:** 2022-03-30

**Authors:** Joanna Bogusiewicz, Magdalena Gaca-Tabaszewska, Dominik Olszówka, Karol Jaroch, Jacek Furtak, Marek Harat, Janusz Pawliszyn, Barbara Bojko

**Affiliations:** 1 Department of Pharmacodynamics and Molecular Pharmacology, Faculty of Pharmacy, Collegium Medicum in Bydgoszcz, Nicolaus Copernicus University in Torun, 85-089 Bydgoszcz, Poland; j.bogusiewicz@cm.umk.pl (J.B.); magda.gaca5@gmail.com (M.G.-T.); dominikolszowka95@gmail.com (D.O.); karol.jaroch@cm.umk.pl (K.J.); 2 Department of Neurosurgery, 10th Military Research Hospital and Polyclinic, 85-681 Bydgoszcz, Poland; jacek.furtak2019@gmail.com (J.F.); harat@10wsk.mil.pl (M.H.); 3 Department of Neurosurgery and Neurology, Faculty of Health Sciences, Collegium Medicum in Bydgoszcz, Nicolaus Copernicus University in Torun, 85-168 Bydgoszcz, Poland; 4 Department of Chemistry, University of Waterloo, Waterloo, ON M1B 6G3, Canada; janusz@uwaterloo.ca

**Keywords:** SPME, CBS-MS, brain tumors, lipidomics

## Abstract

Brain tumors are neoplasms with one of the highest mortality rates. Therefore, the availability of methods that allow for the quick and effective diagnosis of brain tumors and selection of appropriate treatments is of critical importance for patient outcomes. In this study, coated blade spray-mass spectrometry (CBS-MS), which combines the features of microextraction and fast ionization methods, was applied for the analysis of brain tumors. In this approach, a sword-shaped probe is coated with a sorptive material to enable the extraction of analytes from biological samples. The analytes are then desorbed using only a few microliters of solvent, followed by the insertion of the CBS device into the interface on the mass spectrometer source. The results of this proof-of-concept experiment confirmed that CBS coupled to high-resolution mass spectrometry (HRMS) enables the rapid differentiation of two histologically different lesions: meningiomas and gliomas. Moreover, quantitative CBS-HRMS/MS analysis of carnitine, the endogenous compound, previously identified as a discriminating metabolite, showed good reproducibility with the variation below 10% when using a standard addition calibration strategy and deuterated internal standards for correction. The resultant data show that the proposed CBS-MS technique can be useful for on-site qualitative and quantitative assessments of brain tumor metabolite profiles.

## 1. Introduction

Central nervous system (CNS) neoplasms are characterized by wide histological and molecular diversity, a fact that is acknowledged in the World Health Organization’s (WHO) brain tumor classification [1,2]. Meningiomas are a very common type of primary tumor, accounting for approximately 36% of all brain tumors. These neoplasms, which originate in arachnoid cap cells, are mostly benign; however, some become malignant [3]. On the other hand, there is still debate as to whether gliomas, the most common malignant primary brain tumor, originate in the neural stem cells or the glial cells [4]. Around 45% of all gliomas are classified as glioblastomas, which are one of the deadliest neoplasms, with a 5-year relative survival rate of 5% [5]. In general, the most basic approach to treating brain tumors is to first remove the lesion surgically, followed by chemo- and/or radiotherapy. This approach is challenging not only because of the brain’s complex anatomical and functional structures, but also due to the ability of neoplasms to infiltrate healthy cells, as is observed in lesions such as glioblastomas [6]. Furthermore, challenges related to accessing the brain structure make it very difficult to analyze the level of targeted analytes such as biomarkers or administrated drugs in brain tumors. Therefore, it is important to have access to tools that are capable of addressing these problems as they can enable fast intraoperative decisions regarding further steps [1].

Many mass-spectrometry (MS) based approaches have already been introduced for medical applications. Ambient-MS techniques, which omit chromatographic separation, would be even more useful due to their ability to provide results quickly. Desorption electrospray ionization mass spectrometry (DESI-MS) is one such method that enables the rapid imaging of biological tissues. In this method, an electrically charged solvent mist is sprayed onto the surface of a thin specimen, and the charged droplets, along with the ionized analytes, are then redirected into the atmospheric pressure interface, leading to the mass spectrometer [7]. This ex vivo technique enables researchers to visualize the spatial distribution of analytes, which can then be used to differentiate brain tumors from healthy tissue as well as for tumor classification [8,9]. Matrix-assisted laser desorption/ionization (MALDI-MS), which uses a laser to ionize molecules from a laser-energy-absorbing matrix mixed with the sample [10], is another technique that can be used for margin assessment and the analysis of the spatial distribution of targeted analytes. Among the various ambient mass spectrometry techniques, rapid evaporative ionization mass spectrometry (REIMS) should also be mentioned. This technique is based on the analysis of aerosols generated during the cutting of tissue with an electrocautery blade or other tool, which is then directed at the mass spectrometer [11]. This tool, also known as an intelligent knife (iKnife), was widely used in the surgical differentiation of healthy and cancerous tissues [12]. Other techniques mainly used to assess the concentration of analytes in a given sample such as paper spray (PS) and probe electrospray ionization (PESI) entail ionizing the sampled analytes via the application of high voltage to the probe, which is installed in a dedicated interface mounted on the source of the mass spectrometer [10].

Recently, the combination of solid-phase microextraction (SPME) and direct or ambient MS methods such as MOI-MS and CBS-MS have been reported in medical applications [13,14,15]. These techniques harness the features of SPME while also providing fast analysis by eliminating the need for the chromatographic separation process. SPME is based on the interaction between analytes in biological material and sorbent that is coated on the support such as a fiber, blade, or mesh [16]. This approach enables analytes to be extracted from biological matrices while not consuming any of the matrix and withdrawing only small amounts of the targeted molecules. In the CBS-MS approach, analytes are extracted from a biological matrix using a specially designed sword-shaped probe coated with immobilized sorptive particles. After extraction, the probe is inserted into the interface of the MS source, where a drop of desorption solvent is placed on the blade surface to release analytes and high voltage is applied to enable ionization and the analysis of the extracted substances [17,18].

Taking into consideration the challenges associated with neuro-oncology and the features of SPME-based ambient mass spectrometry technologies, this proof-of-concept study aims to assess the suitability of CBS-MS for analyzing endogenous metabolites in brain tumors—qualitatively via untargeted lipidomic profiling and quantitatively by calculating the concentrations of the selected analytes.

## 2. Results and Discussion

**Figure 1:**
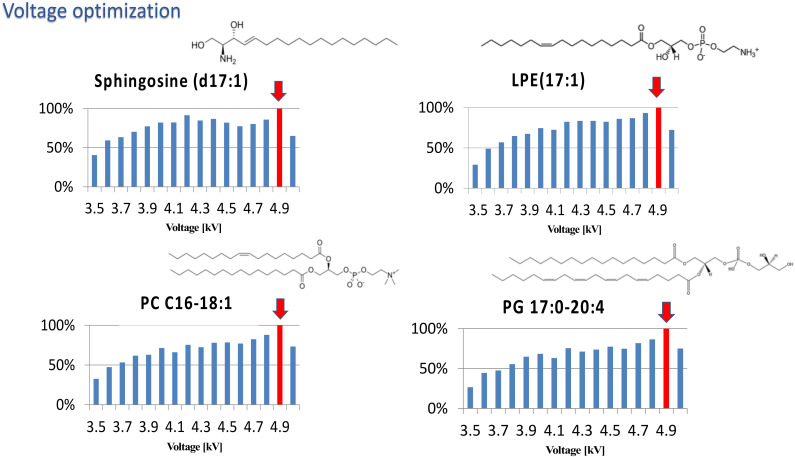
Exemplary plots showing the optimization of voltage for untargeted lipidomic analysis with use of IPA:MeOH, 1:3, *v*/*v* with 10 mM ammonium acetate and 1 mM acetic acid. Four analytical standards were used to optimize the desorption solvent: PG (17:0–20:4), PC (C16–18:1), LPE (17:1), and Sphingosine (d17:1). The red arrows indicate voltage selected for further experiments.

**Figure 2:**
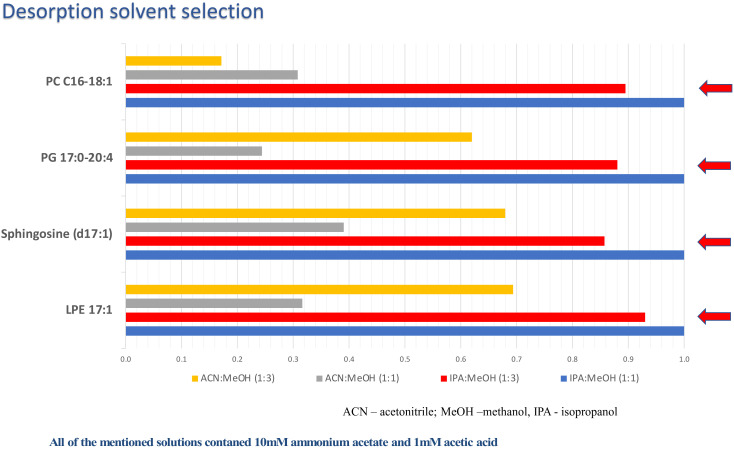
Plots presenting the optimization of desorption solvent for untargeted lipidomic analysis. Four analytical standards were used to optimize the desorption solvent: PG (17:0–20:4), PC (C16–18:1), LPE (17:1), and Sphingosine (d17:1). Acetonitrile:methanol (1:1), acetonitrile:methanol (1:3), isopropanol:methanol (1:1), and isopropanol:methanol (1:3) solutions all contained 10 mM ammonium acetate and 1 mM acetic acid was tested as a desorption solution. The red arrows indicate the desorption solvent selected for further experiments.

Ambient mass spectrometry methods can be an invaluable tool in the operating room as they enable the fast analysis of biological materials, which can in turn guide surgeons in their approach. Many of these methods enable the differentiation of cancerous and healthy tissue as well as the classification of tumors based on their histological and molecular features. Since such differentiation is often based on lipid profiling [8,9,19,20,21], the CBS-MS platform was applied to perform untargeted lipidomic analyses of brain tumors. Nevertheless, before sample analysis, the applied voltage and the desorption solvent were optimized. For this purpose, four mixtures of solvents spiked with lipid standards were tested. Then, the voltage in the range of 3.5 to 5.0 kV was tested, peak areas for spiked lipids at a given voltage were noted, and optimal voltage was chosen based on the highest peak area. An exemplary plot is presented in Figure 1. To select the best desorption solvent, peak areas of the spiked standard were compared (Figure 2). Finally, the mixture of isopropanol:methanol, IPA:MeOH (1:3, *v*/*v*) with 10 mM ammonium acetate and 1 mM acetic acid was chosen. However, it should be noted that both IPA:MeOH, 1:3, *v*/*v* with 10 mM ammonium acetate and 1 mM acetic acid and IPA:MeOH, 1:1, *v*/*v* with 10 mM ammonium acetate and 1 mM acetic acid were characterized by similar peak areas; nevertheless, relative standard deviation was higher for the second mixture (Table S1).

Next, CBS probes were inserted into two types of tumors with different histological origins: meningiomas and gliomas. Since cancerous lesions can be characterized by high heterogeneity, another matrix such as plasma was used as the homogenous quality control [22]. Thus, the obtained lipidomic data were filtered based on the coefficient of variation values of the analytes present in plasma (RSD < 30 but not equal to 0). Although the use of this matrix as a QC could lead to the loss of analytes that were not present in plasma, this approach provides a good trade-off between coverage and quality.

**Figure 3:**
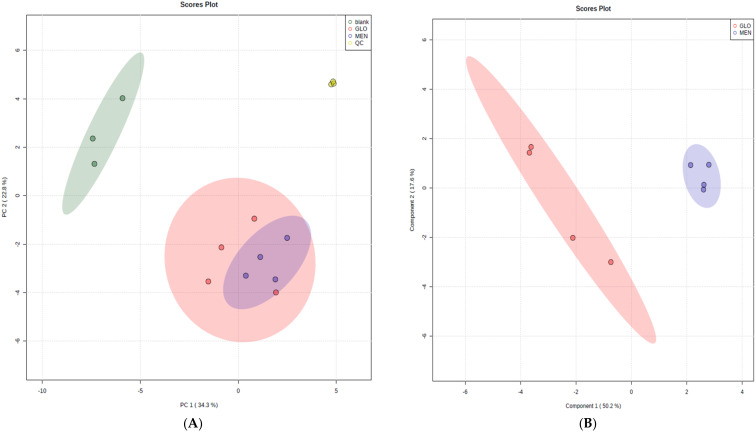
(**A**) Principal component analysis of brain tumors based on lipidomic analysis. The PLS-DA can be found in the Supplementary Materials (Figure S1A). (**B**) Partial least squares analysis of meningiomas and gliomas. The PCA data can be found in the Supplementary Materials (Figure S1B).

Principal component analysis (PCA) of the studied groups revealed clear separation between the extraction blanks, plasma, and tumors (Figure 3A). The plasma samples formed a very tight cluster, which showed that the instrumental analysis for the selected analytes was reproducible. Moreover, further partial least squares analysis performed on a set of tentative lipids with VIP-scores above 1.0 enabled the differentiation of two histologically distinct lesions: meningiomas and gliomas (Figure 3B). The R^2^s for the one and two components were 0.89 and 0.99, respectively, while the Q2 values were 0.72 and 0.76, respectively. These results indicate that CBS-MS technology can be as useful in the differentiation of brain tumors as other MS-based methods such as REIMS or DESI-MS. The main advantage of CBS-MS is that it does not consume any of the tissue upon sampling, which allows the sample to be used for other analyses such as immunochemistry. REIMS, on the other hand, enables the analysis of aerosols generated during the cutting of a tumor either in real-time (in vivo characterization of the studied tissue) or after resection. Notably, further complementary analysis can be performed on the intact region of the specimen not affected by the iKnife. Similar to CBS-MS, DESI-MS allows for the repetitive analysis of the same sample; however, it is necessary to cut the specimen into thin slices prior to instrumental experimentation, which prolongs the pretreatment step and limits intraoperative application. In contrast, SPME-based technologies are based on a simple protocol, which allows them to be easily implemented by surgery room personnel [23].

For routine diagnostics, the most common approach is to determine the concentration of target analytes (i.e., biomarkers). Therefore, in the next step, CBS-MS was applied to assess the levels of the exemplary metabolite, carnitine, in a glioma. This analyte was selected due to its usefulness in discriminating between different grades of tumor, as was demonstrated in Goryńska et al.’s [24] recent work with SPME-LC-HRMS. Moreover, the relationship between changes in the levels of carnitine and its esters, acylcarnitines, and tumor grades has been documented elsewhere [25,26,27]. These analytes play an important role in fatty acid metabolism, and consequently, the composition of the lipidome [25,26]. Therefore, using a fast method such as CBS-MS for the quantitative analysis of carnitine can be highly valuable, as shown by the proof-of-concept study documented herein. The CBS probe is rather unsuited for in vivo analysis as it lacks the low-invasiveness of fiber-based SPME; however, it is very useful as a tool for rapid instrumental analysis during intra-operative diagnostics. As demonstrated, CBS probes can be used successfully to sample intact resected tumors on-site; however, the calibration of the target endogenous metabolites remains a challenge. Thus, the results of two calibration approaches were compared for homogenized tissue and an intact tumor.

**Figure 4:**
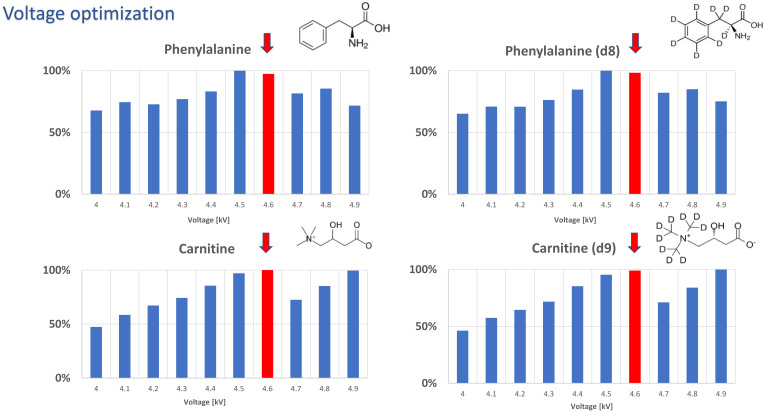
Exemplary plots showing the optimization of voltage for targeted analysis with the use of ACN:H_2_O, 95:5, *v*/*v*. Four analytical standards—phenylalanine, phenylalanine(d8), L-carnitine inner salt, L-carnitine(trimethyl-d9)—were used to optimize the desorption solvent: The red arrows indicate the voltage selected for further experiments.

**Figure 5:**
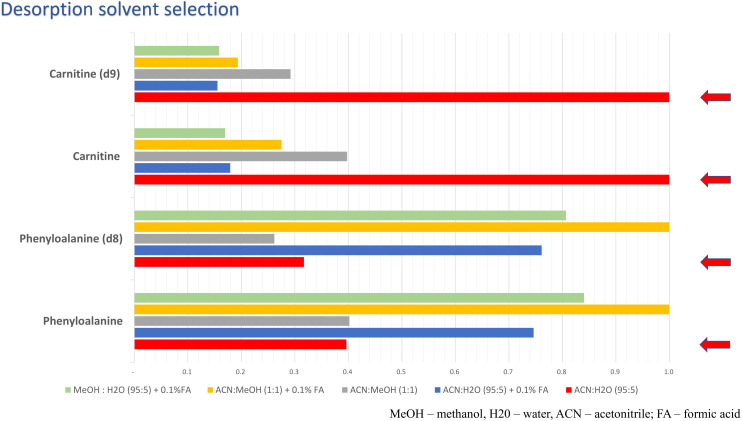
Plots presenting the optimization of the desorption solvent for targeted analysis. Four analytical standards were used to optimize the desorption solvent: phenylalanine, phenylalanine(d8), L-carnitine inner salt, L-carnitine(trimethyl-d9). Methanol:water (95:5) + 0.1% formic acid, acetonitrile:methanol (1:1) + 0.1% formic acid, acetonitrile:methanol (1:1), acetonitrile:water (95:5) + 0.1% formic acid and acetonitrile:water (95:5) was tested as a desorption solution. The red arrows indicate the desorption solvent selected for further experiments.

Prior to the analysis of real samples, the optimal voltage and desorption solvent were selected. Thus, five different desorption solvents spiked with L-carnitine(trimethyl-d9), L-carnitine, phenylalanine, and phenylalanine(d8) were tested. The voltage range of 4.0 to 4.9 kV was studied and the results of voltage optimization for acetonitryle:water, 95:5, *v*/*v* solution as an exemplary plot are presented in Figure 4. To select the best desorption solvent for carnitine, peak areas of the spiked standard were compared (Figure 5) and an acetonitrile:water (95:5) mixture was selected as the optimal solution for carnitine analysis.

In the first approach, a calibration curve was constructed for a deuterated internal standard (IS) spiked in homogenized tumor tissue, while the second approach was based on standard addition. In the second approach, two calibration curves were constructed, one using the raw data and the other one using peak areas normalized by an internal standard (IS) added to the homogenate (Table S2). The formula and the coefficient of determination (R^2^), which was above 0.9 for both curves, are provided in Figure S2. The concentration of targeted compounds in the studied tumor was 65.7 9 ± 25.66 µg/mL based on the formula obtained from raw data, while calculations based on normalized data yielded a higher value of 100 ± 10 µg/mL. In both cases, the dilution factor of homogenate with phosphate buffered saline (PBS) was taken into account to calculate the concentration in the tumor. This result indicates that IS should be added whenever available as it significantly improves the quality of the data. Since neither the human metabolome database (HMDB0000062) nor the available literature contained unified data for carnitine concentrations in brain tumors also in terms of the applied methodology, no head-to-head comparison with the published literature could be made [28,29]. However, the order of magnitude is in concordance with the data reported by Miyata et al. [28]. As this is a proof-of-concept study performed only on one brain tumor, high inter-tumoral variability can be expected based on the high heterogeneity of these neoplasms [22].

**Figure 6:**
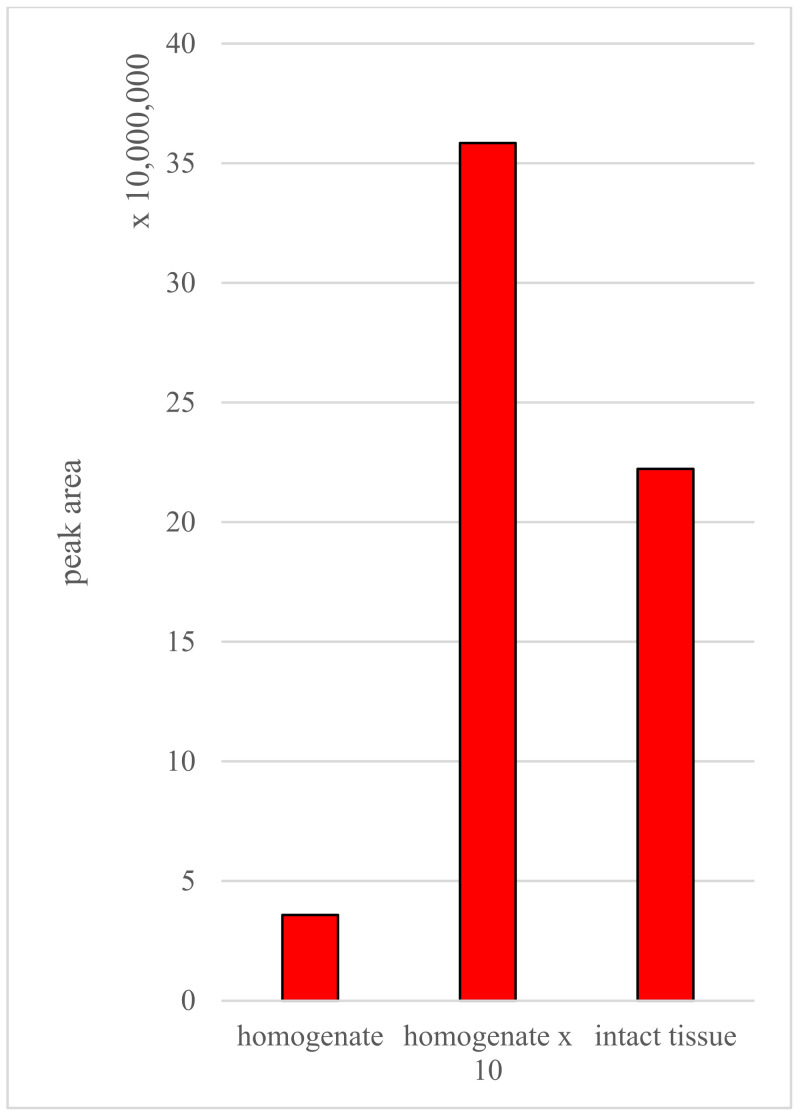
Peak area for carnitine in an exemplary sample of the homogenate and in intact tissue.

Next, the peak areas of carnitine obtained for the homogenate sample were compared with those of the intact tissue (Figure 6). The highest peak was observed in the homogenate sample, while the values obtained for the intact tissue were generally lower. This phenomenon may be due to the release of metabolites during the homogenization procedure. It is important to note that matrix-matched calibration for SPME enables the calculation of the free concentration of the studied analytes (i.e., not bound to biomolecules such as lipoproteins or proteins), while a matrix-free approach (i.e., with the use of agarose gel) permits the measurement of the obtained total concentration. However, the release of intracellular/intercompartmental metabolites during homogenization increases the free fraction, which thus becomes available to the SPME probes. Consequently, the amount of carnitine in the intact tissue calculated based on the matrix-matched calibration curve can be considered relative to homogenized tissue. In contrast, the concentration of carnitine in the intact tissue calculated based on the calibration curve prepared with L-carnitine(trimethyl-d9) in homogenate (Figure S3) was 73.57 µg/mL, with a LOD and LOQ of 16.60 µg/mL and 50.33 µg/mL, respectively. Although the relative concentration obtained from the intact tissue corresponded to the concentration measured in the homogenate (calculated based on the standard addition method), several issues should be discussed prior to further attempts to develop the calibration strategy to determine endogenous compounds in cancer tissue with CBS-MS. First, since brain tumors are characterized by high heterogeneity, the homogenization of a small section may only result in the selection of a portion of the tumor’s phenotype [22]. Conversely, SPME probes enable the extraction of analytes along the sorbent from various parts of the tumor [30]. Second, the CBS-MS device has a bigger extraction surface than SPME fibers, which means that it can be influenced more easily by the composition of a changed region (e.g., the calcified part of the tumor where extraction is altered at a specific part of the blade).

Finally, it should be noted that the use of optimal parameters is crucial in ensuring good data quality during the targeted analysis of metabolites or potential biomarkers. For example, although carnitine was the main focus of the current study, phenylalanine concentration was also assessed as it offers good discriminative power in the differentiation of gliomas with and without (i.e., wildtype) IDH mutation as well as in the stratification of malignancy grade [24]. The findings showed increased levels of phenylalanine in cancerous cells, which is related to the higher demand for this amino acid in neoplastic cells. In fact, the injection of phenylalanine derivatives such as 8F-fluorodihydroxyphenylalanine (18F-DOPA) has been employed for tumor visualization and differentiation [31]. As such, similar to the determination of carnitine, the standard addition method with and without correction with deuterated IS was applied (Table S3). The formulas and coefficients of determination (R^2^) are shown in Figure S4. As can be seen, the plot constructed using the raw data was unacceptable (R^2^ = 0.31), while the same parameters for the normalized data were equal to 0.996. Apart from a high R^2^, very high variations were observed for the raw data (Table S3). This result further confirms the importance of employing internal standards whenever possible. The high RSD could be related to the low concentration of the analyte in this particular tumor compared to later data obtained from the “single-point” screening of different lesions; however, it was more likely to be caused by the low compatibility of the desorption solvent, which is one of the most crucial parameters in the method optimization protocol. As detailed previously (Figure 4 and Figure 5), the mixture used in the experiment was suboptimal for phenylalanine. On the other hand, this observation indicates that untargeted analysis does not ensure good quality data along with the entire range of detected metabolites and, as such, may not enable the quantitative analysis of all compounds of interest under the “general” conditions. However, as demonstrated in the current work, untargeted profiling via CBS can allow rapid screening for the selection of discriminating metabolites. Then, targeted quantitative analysis with analyte-specific conditions to increase efficiency could be applied.

## 3. Materials and Methods

### 3.1. Chemicals and Materials

The liquid chromatography-mass spectrometry (LC-MS) grade solvents (i.e., isopropanol, methanol, water, and acetonitrile) and additives (i.e., ammonium acetate, acetic acid, and formic acid) used in this work were purchased from Sigma Aldrich (Poznan, Poland). The probes were prepared using N, N-dimethylformamide ACS reagent and polyacrylonitrile. In addition, the phosphate-buffered saline used in the experiments was acquired from Sigma Aldrich (Poznan, Poland).

The standards selected for this work included Sphingosine (d17:1), LPE (17:1), PC (C16–18:1), PG (17:0–20:4), L-carnitine(trimethyl-d9), L-carnitine inner salt, phenylalanine, and phenylalanine(d8). The lipid short-hand notation presented herein was used in this manuscript.

Coated blade spray-mass spectrometry probes (CBS-MS) were prepared according to a procedure described elsewhere [18]. Two types of sorbents were used in the experiments: octadecyl (C18), which was purchased from Anchem (Toruń, Poland), and hydrophilic–lipophilic balance particles (HLB), which were manufactured and kindly provided by the University of Waterloo [32].

### 3.2. Biological Material

Nine brain tumors (five gliomas, four meningiomas) were excised during neurosurgical procedures at the 10th Military Research Hospital and Polyclinic in Bydgoszcz. Four meningiomas and four gliomas were used in the first untargeted lipidomic experiment, while the remaining tumor (glioma) was used in the assessment of the concentration of selected metabolites. Directly after removal, the tumors were transported to the laboratory in a Styrofoam box filled with ice packs and stored at −30 °C until sampling and instrumental analysis.

The study was approved by the Bioethical Committee in Bydgoszcz (KB 628/2015).

### 3.3. Instrumental Analysis

All experiments were carried out using a Q Exactive Focus mass spectrometer (Thermo Scientific, Bremen, Germany). To perform CBS-MS analysis, a special interface was installed on the mass spectrometer source. The interface was kindly provided by the University of Waterloo, where it was manufactured. The mass spectrometry parameters were optimized for each experiment separately and are provided in the following subsections.

### 3.4. General CBS-MS Protocol

**Figure 7:**
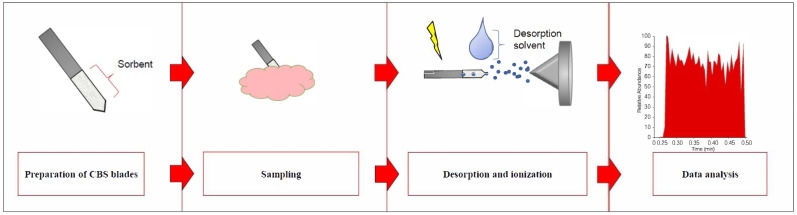
Workflow of the coated blade spray-mass spectrometry (CBS-MS).

CBS probes with a coating length of 1 cm were used in all experiments. Probes coated with one of two different sorbents were used depending on the type of analysis; specifically, a C18 coating was used for untargeted lipidomic screening, and an HLB coating was used for the targeted analysis of selected metabolites. To remove any possible impurities from the probes (e.g., residue from the blade-preparation process), they were placed in a methanol:acetonitrile: isopropanol, (50:25:25 *v*/*v*/*v*) solution and agitated on a Benchtop (Multi-Tube Vortexer) shaker for 30 min. The sorbent was then activated by placing the probes in a methanol:water (1:1 *v*/*v*) solution, with agitation at 1200 rpm for 75 min. The general workflow of the proposed CBS-MS approach was similar for all conducted experiments and is detailed in the following subsections (Figure 7).

#### 3.4.1. Untargeted Lipidomic Analysis

The brain tumors were thawed and divided into two groups: meningiomas and gliomas.

Next, C18-coated CBS probes were inserted to conduct a 15-min extraction. After the extraction time had elapsed, the blades were removed from the tissue and rinsed with water to remove any remaining matrix components and unspecific bound proteins. After rinsing, the CBS probes were installed in the special interface mounted on the MS, and a 10 μL mixture consisting of isopropanol + methanol (1:3) + 10 mM ammonium acetate + 1 mmol acetic acid was added to the surface of the blade to desorb the analytes. Desorption was conducted for 60 s, followed by the application of 4.9 kV voltage to the CBS device for 30 s. All experiments were performed on a mass spectrometer in full-scan mode (mass range: 1,200,000–10,000,000), with a capillary temperature of 320 °C and an S-lens level at 50 V.

To increase the reliability of the obtained data, quality control prepared by sampling the plasma samples of the patients with both gliomas and meningiomas was used. This material was chosen due to its homogeneity independent of the tumor type. Apart from the QC sample, the sequence contained blanks obtained by the application of the whole CBS-MS protocol with the omission of sampling steps. Blanks and QC samples were run every four samples and all tumor samples were randomized across the sequence.

#### 3.4.2. Targeted Analysis of Selected Metabolites

These experiments were performed using glioma tumor tissue and its homogenate, which was produced by homogenizing 700 mg of the tissue with 7 mL PBS in a BeadBug microtube homogenizer (Benchmark) for 2 min at 4000 rpm using 3.0 mm zirconium beads. Then, the homogenate was then divided into two main portions. The first portion was used for the assessment of carnitine and phenylalanine concentrations via the standard addition method. For this purpose, the homogenates were spiked with 25 µg/mL of internal standards (2-L-carnitine(trimethyl-d9), phenylalanine(d8)) and analyte at a set concentration (Tables S2 and S3). Apart from the standard addition curve, calibration curves were prepared for 2-L-carnitine(trimethyl-d9). The concentration levels of 2-L-carnitine(trimethyl-d9) were 10 µg/mL, 20 µg/mL, 40 µg/mL, and 50 µg/mL. Subsequently, in all experiments, 20 μL of homogenate was spread evenly on the blade followed by a 15-min extraction, and another CBS probe was inserted into the intact glioma sample for 15 min. After the extractions had been completed, the homogenate was wiped off with a Kimwipe and the probes were washed for 5 s with water to remove any biological residuals. Next, the CBS probes were placed into the interface on the mass spectrometer for a 45 s desorption with 20 μL of an acetonitrile/water (95:5 *v*/*v*) mixture, followed by ionization and the introduction of the analytes to MS (4.6 kV for 15 s). All experiments were performed on a Q Exactive Focus mass spectrometer in full-scan mode (scan range: 500,000–7,500,000) with MS/MS confirmation of masses from the inclusion list of the studied analytes. The capillary temperature was set at 320 °C, with S-lenses of 50 V. Finally, the fragmentation parameters were as follows: mass resolution—35,000 FWHM; AGC target—5 × 10^4^; minimum AGC—8 × 10^3^; intensity threshold—auto; maximum IT—auto; isolation window—3.0 *m*/*z*; normalized stepped collision energy—20 V, 30 V, 50 V; loop count—1; and dynamic exclusion—auto.

### 3.5. Data Processing and Statistical Analysis

Data acquisition was performed using Xcalibur v. 4.2 software (Thermo Fisher Scientific, San Jose, CA, USA). The data for the lipidomic studies were processed using LipidSearch v. 4.1.30 software (Thermo Fisher Scientific, San Jose, CA, USA), with the accuracy set to 3 ppm and the intensity threshold set to 100,000. The searched ion adducts consisted of H^+^ and NH_4_^+^. An m-score of 10 and a retention time tolerance of 0.25 min. were used as the alignment settings and based on extraction quality control (QC), and the results were filtered using the following parameters: a coefficient of variation (CV) below 30 and not equal to 0. After the results had been filtered, the peak areas for all tentatively detected lipids were normalized on the summary peak area. Multivariate statistical analysis was performed using the online Metaboanalyst software package [33], with missing values being estimated and replaced by small numbers (i.e., half of the minimum positive values in the study data). Logarithmic transformation and autoscaling were also applied. Finally, two multivariate approaches, principal component analysis (PCA) and partial least squares discriminant analysis (PLS-DA), were also employed. The selection of lipids for PLS-DA analysis was based on a VIP score greater than 1.0.

The concentration of carnitine calculated based on standard addition methods was given as a concentration +/– relative standard deviation (RSD). Additionally, to estimate the level of carnitine in the intact tissue, the discrepancy in the intensities of the deuterated and non-deuterated standards of carnitine was compared during liquid chromatography-mass spectrometry analysis (LC-HRMS). The peak area for the deuterated IS was 20% lower than for the non-deuterated standard at the same concentration (Figure S5). Therefore, the concentration of intact tissue was recalculated accordingly.

The limit of detection (LOD) and limit of quantification (LOQ) were calculated using the formulas, LOD = [3.3 × (σ/s)] and LOQ = [10 × (σ/s)], where “σ” is considered the response and “s” is considered to be the slope of the calibration curve.

## 4. Conclusions

Coated blade spray-mass spectrometry is a simple and fast technology that enables results in only a few minutes. The present work has shown that this methodology can be applied successfully not only for the lipidomic differentiation of meningiomas and gliomas, but also for the quantitative analysis of carnitine. Although this study was conducted entirely in the university facility, the results indicate that the proposed CBS-MS method could be applied on-site in clinical environments in the future, largely due to CBS’s user-friendly extraction protocol.

## Data Availability

Not applicable.

## Supplementary Materials

The following supporting information can be downloaded at: https://www.mdpi.com/article/10.3390/molecules27072251/s1, Figure S1. (A) Partial least squares data analysis (PLS-DA) of the studied samples based on tentative lipids. (B) Principal component analysis of meningiomas and gliomas based on tentative lipids with a VIP-score above 1.0; Figure S2. Standard addition curve of carnitine; (A) raw data; (B) -data normalized on internal standard (IS) area; Figure S3. Calibration curve for carnitine(trimethyl-d9) in brain tumor homogenate; Figure S4. Standard addition curve of phenylalanine; (A) raw data; (B) -data normalized on internal standard (IS) area; Figure S5. Comparison of the peak areas of the mixture of carnitine and its deuterated form in the concentration 10 ppm; Table S1. Peak areas and RSD for lipids used in the selection of desorption solvent for untargeted analysis; Table S2. Standard addition curve details for carnitine; Table S3. Standard addition curve details for phenylalanine. Reference [34] are cited in the supplementary materials.

## Sample Availability

Samples of the compounds are not available from the authors.
