# Supplementary material for: Coated Blade Spray-Mass Spectrometry as a New Approach for the Rapid Characterization of Brain Tumors
Source: Molecules. 2022 Mar 30;27(7):2251. doi: 10.3390/molecules27072251 (PMC9000701; doi:10.3390/molecules27072251)
Supplement: Supplementary file 1 [file molecules-27-02251-s001.zip › molecules-1651104-supplementary.pdf]

Coated Blade Spray-Mass Spectrometry as a New Approach for the Rapid Characterization of Brain Tumors

Joanna Bogusiewicz[1,†], Magdalena Gaca-Tabaszewska[1,†], Dominik Olszówka[1], Karol Jaroch[1], Jacek Furtak[2], Marek Harat[2,3], Janusz Pawliszyn[4] and Barbara Bojko[1*]

1 Department of Pharmacodynamics and Molecular Pharmacology, Faculty of Pharmacy, Collegium Medicum in Bydgoszcz, Nicolaus Copernicus University in Torun, 85-089 Bydgoszcz, Poland, j.bogusiewicz@cm.umk.pl (J.B.); magda.gaca5@gmail.com (M.G.T.); dominikolszowka95@gmail.com (D.O.); karol.jaroch@cm.umk.pl (K.J.); bbojko@cm.umk.pl (B.B)

2 Department of Neurosurgery, 10th Military Research Hospital and Polyclinic, 85-681 Bydgoszcz, Poland; jacek.furtak2019@gmail.com (J.F.); harat@10wsk.mil.pl (M.H.)

3 Department of Neurosurgery and Neurology, Faculty of Health Sciences, Collegium Medicum in Bydgoszcz, Nicolaus Copernicus University in Torun, 85-168 Bydgoszcz, Poland

4 Department of Chemistry, University of Waterloo, Waterloo, ON M1B 6G3, Canada; janusz@uwaterloo.ca (J.P.)

* Correspondence: bbojko@cm.umk.pl; Tel.: +48-525-853-564

† These authors contributed equally

SUPPLEMENTARY MATERIALS

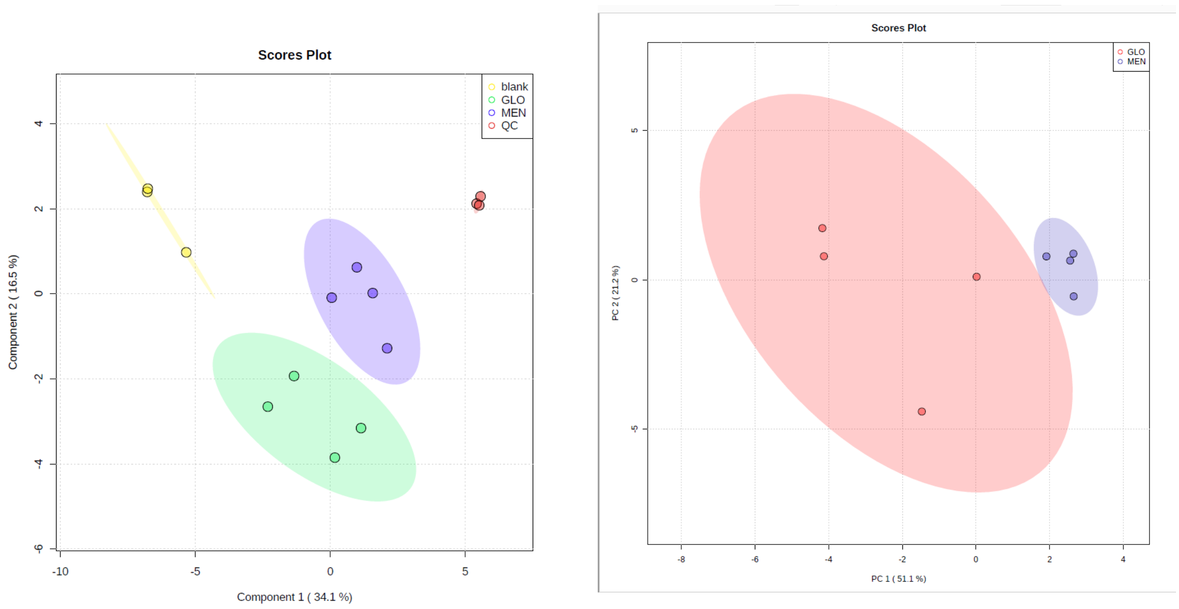

(A) (B)

Figure S1. (A) Partial least squares data analysis (PLS-DA) of studied samples based on tentative lipids. Model passed permutation test with three components. R2 were 0.98 and Q2 at level 0.70; (B) Principal component analysis of meningiomas and gliomas based on tentative lipid with VIP-score above 1.0.

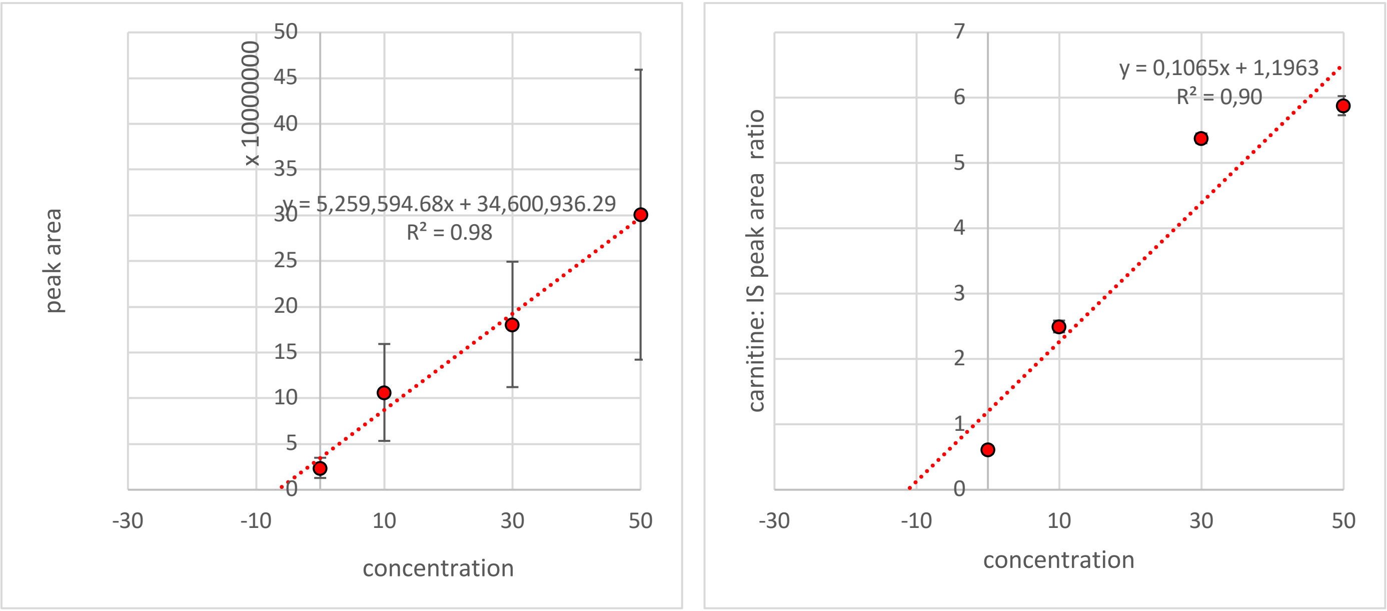

(A) (B)

Figure S2. Standard addition curve of carnitine; (A) raw data; (B)- data normalized on internal standard (IS) area. Bars represent the standard deviation of peak areas and carnitine: IS peak area ratios.

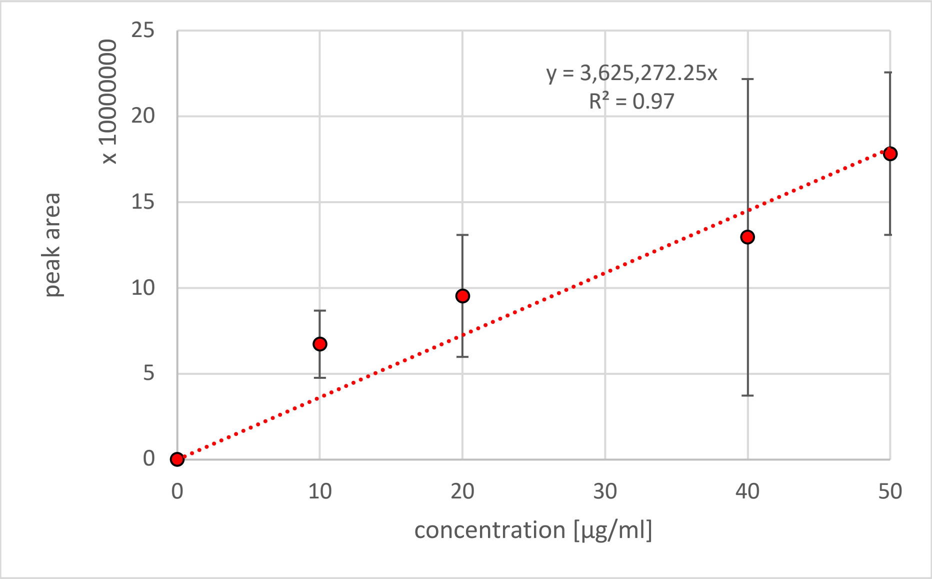

Figure S3. Calibration curve for carnitine(trimethyl-d9) in brain tumor homogenate. Bars represent the standard deviation of peak areas

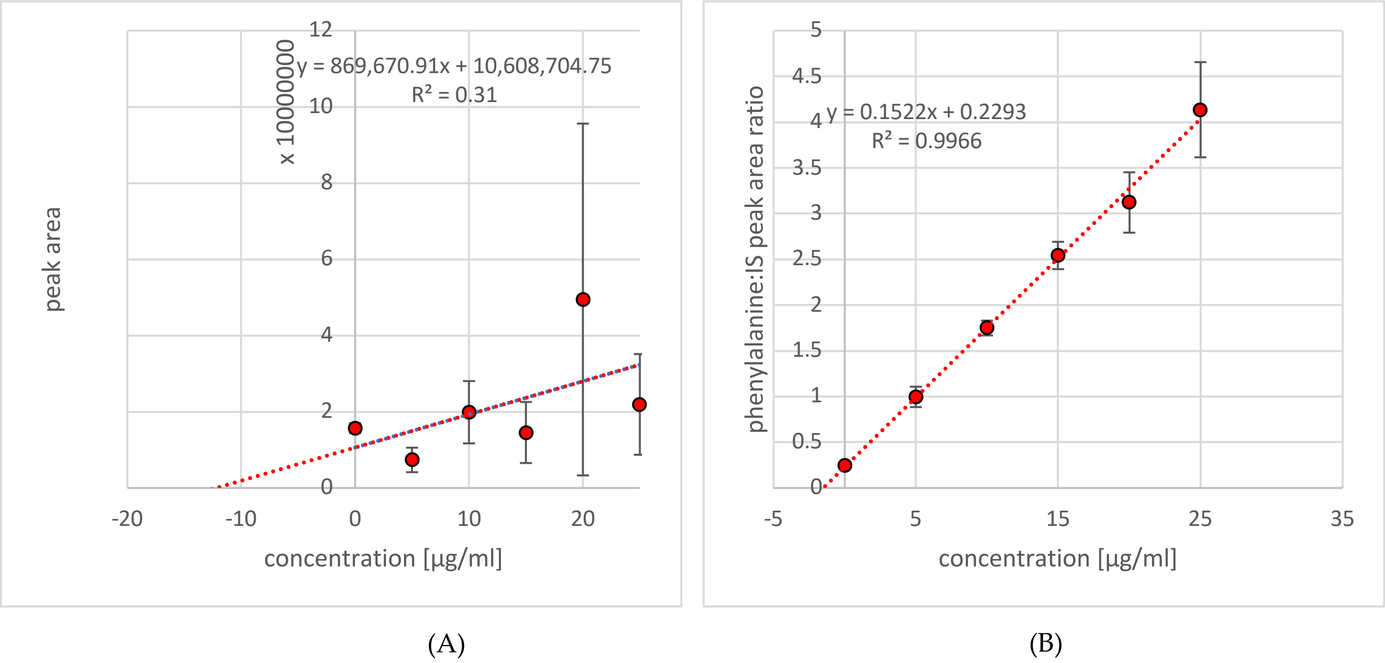

Figure S4. Standard addition curve of phenylalanine; (A) raw data; (B)- data normalized on internal standard (IS) area. Bars represent the standard deviation of peak areas and phenylalanine: IS peak area ratios.

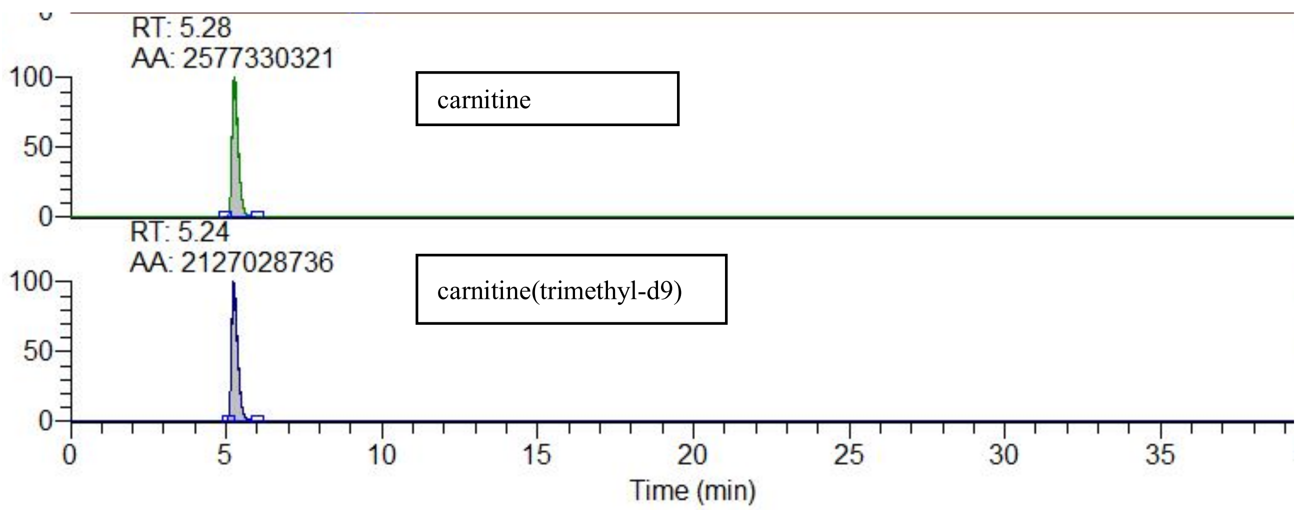

Figure S5. Comparison of the peak areas of the mixture of carnitine and its deuterated form in the concentration 10 ppm. The methodology of this analysis was presented in Bogusiewicz et al. (Bogusiewicz et al. 2020)* under metabolomics analysis on PFP column.

** Bogusiewicz, J.; Goryńska, P.Z.; Gaca, M.; Chmara, K.; Goryński, K.; Jaroch, K.; Paczkowski, D.; Furtak, J.; Harat, M.; Bojko, B. On-Site Sampling and Extraction of Brain Tumors for Metabolomics and Lipidomics Analysis. J. Vis. Exp. 2020, 2020, 159, doi:10.3791/61260.*

Table S1. Peak areas and RSD for lipids used in the selection of desorption solvent for untargeted analysis.

sample [x] + phenylalanine	IPA:MeOH,1:3,v/v with 10 mM ammonium acetate and 1 mM acetic acid		IPA:MeOH,1:1,v/v with 10 mM ammonium acetate and 1 mM acetic acid	
	**Average area**	**RSD**	**Average area**	**RSD[1]**
PC C16-18:1	811259	23%	766728	51%
LPE 17:1	939323	9%	1000845	38%
PG 17:0-20:4	474606	28%	493471	53%
Sphingosine (d17:1)	2008763	23%	7106255	110%

[1] relative standard deviation

Table S2. Standard addition curve details for carnitine

Sample [x] + carnitine	Raw data		Normalized data	
	**area**	**RSD**	**ratio**	**RSD[1]**
x+50 ug/ml	300725268	62%	5.88	3%
x+30 ug/ml	428917804	46%	5.38	1%
x+10 ug/ml	106366685	59%	2.50	4%
X	23917467	39%	0.61	10%

[1] relative standard deviation

Table S3. Standard addition curve details for phenylalanine

sample [x] + phenylalanine	Raw data		Normalized data	
	**area**	**RSD**	**ratio: standard/Deuter**	**RSD[1]**
x+25 ug/ml	21928862	60%	4.14	13%
x+20 ug/ml	49460652	93%	3.12	11%
x+15 ug/ml	14558740	55%	2.54	6%
x+10 ug/ml	19881108	41%	1.75	5%
x+5 ug/ml	7364715	44%	1.00	11%
X	3023055	43%	0.24	13%

[1] relative standard deviation
